# Critical review: Pérez Peña E, ed. Atención integral de la Infertilidad. Endocrinología, Cirugía y Reproducción Asistida. 4 edición. México: Editorial Médica Panamericana; 2020

**DOI:** 10.5935/1518-0557.20200013

**Published:** 2020

**Authors:** Maria do Carmo Borges de Souza

**Affiliations:** 1 Fertipraxis - Centro de Reprodução Humana, Rio de Janeiro, Brazil; 2 Latin American Network of Assisted Reproduction (REDLARA)

## Author Credentials

Efraín Pérez Peña, Mexican, has a long career, initially in Gynecology and Obstetrics, quickly added by his interest in the areas of Reproduction Biology and Endocrinology. He is the author of previous books on infertility that accompany his involvement with the academic field. In Latin America, the use of previous editions of this work is already well known, as well as his incredible hard work and commitment in the organization of the Manual of Assisted Reproduction Procedures of the Latin American Network (REDLARA). Efraín is also responsible for the Education Committee of REDLARA. Now we welcome this fourth edition, dedicated to OBGyn resident physicians and to those who think of specializing in reproductive medicine, but surely also to current specialists. Efraín's striking trajectory is already a beautiful invitation to read this book.

## Abstract

The book has 1011 pages, divided into 50 chapters, written in Spanish. On the one hand, it has a strong content ranging from basic diagnostic aspects of female and male infertility, different etiologies, relevant endocrine aspects such as polycystic ovaries and premature ovarian insufficiency, tubal microsurgery and endometriosis. It explores assisted reproduction from low to high complexity, types of treatment, fertility preservation, related genetics, ethical aspects and boundaries. On the other hand, it also addresses the issues of puberty, sexual differentiation, contraception, endocrinology of pregnancy, the transition to menopause and the knowledge of evidence-based medicine that must be observed.

## Appreciation

David Meldrum's preface to the second edition of this book points out that "it would be a good source of consultation for American fellows, if not the language." For us Latin Americans, Spanish is a common language or, even in Brazil, we have grown accustomed to this interaction, because of REDLARA. Reading the chapters is easy, there is clarity, objectivity and the text is very friendly. The chapters are well referenced if one desires to go more in depth. The presentation of the work is extremely well cared for, it reflects the author in everything.

The use of other specialists in more specific areas greatly enhances the final product, highlights teamwork and strengthens the accuracy and timeliness of content. I greatly appreciated the featured Introduction to each chapter, as a summary of the content, acting as an invitation to reading.

The chapter on the neuroendocrine factor, a well-presented review of hypothalamus-pituitary-gonadal interactions, including clinical features that makes easy reading. Ethical issues deserves special attention due to the interfaces with legal aspects of oocyte donation, replacement uterus and even adoption.

The story of assisted reproduction in the world and in Latin America is visited, with an emphasis on Mexico, of course. The author emphasizes his personal view, as well as the limitations sometimes existing in some geographical information, but he undoubtedly praises the example and persistence of the pioneers, who overcame obstacles that sometimes seemed insurmountable at a certain moment in time of scientific knowledge.

Finally, it is important to highlight the multiple-choice questions at the end of each chapter, which enables self-assessment. It is nice to those who are going to provide some kind of contest. Let us also emphasize the possibility of downloading book content on computers or mobile phones, which brings a very current approach to readers.

